# PLCγ1 inhibition‐driven autophagy of IL‐1β‐treated chondrocyte confers cartilage protection against osteoarthritis, involving AMPK, Erk and Akt

**DOI:** 10.1111/jcmm.16245

**Published:** 2020-12-28

**Authors:** Xiaolei Chen, Yue Wang, Ning Qu, Bing Zhang, Chun Xia

**Affiliations:** ^1^ Zhongshan Hospital Xiamen University Xiamen China; ^2^ School of Medicine Xiamen University Xiamen China

**Keywords:** autophagy enhancement, cartilage damage, ECM synthesis, IL‐1β‐treated chondrocyte, PLCγ1 inhibition, rat OA model

## Abstract

Previous studies identified the involvement of phosphoinositide‐specific phospholipase C (PLC) γ1 in some events of chondrocytes. This study aims to investigate whether and how PLCγ1 modulates autophagy to execute its role in osteoarthritis (OA) progression. Rat normal or human OA chondrocytes were pretreated with IL‐1β for mimicking or sustaining OA pathological condition. Using Western blotting, immunoprecipitation, qPCR, immunofluorescence and Dimethylmethylene blue assays, and ELISA and transmission electron microscope techniques, we found that PLCγ1 inhibitor U73122 enhanced Collagen II, Aggrecan and GAG levels, accompanied with increased LC3B‐II/I ratio and decreased P62 expression level, whereas autophagy inhibitor Chloroquine partially diminished its effect. Meanwhile, U73122 dissociated Beclin1 from Beclin1‐IP3R‐Bcl‐2 complex and blocked mTOR/ULK1 axis, in which the crosstalk between PLCγ1, AMPK, Erk and Akt were involved. Additionally, by haematoxylin and eosin, Safranin O/Fast green, and immunohistochemistry staining, we observed that intra‐articular injection of Ad‐shPLCγ1‐1/2 significantly enhanced Collagen and Aggrecan levels, accompanied with increased LC3B and decreased P62 levels in a rat OA model induced by anterior cruciate ligament transection and medial meniscus resection. Consequently, PLCγ1 inhibition‐driven autophagy conferred cartilage protection against OA through promoting ECM synthesis in OA chondrocytes in vivo and in vitro, involving the crosstalk between PLCγ1, AMPK, Erk and Akt.

## 1 INTRODUCTION

Osteoarthritis (OA) is a degenerative joint disease, characterized by deterioration in the integrity of cartilage, including the loss of cells and abnormal remodelling of cartilage extracellular matrix (ECM).^1^ As the only cell type found in mature cartilage, the chondrocyte gradually becomes a central target for therapeutic manipulation, as dysregulated chondrocyte physiology dominates the initiation and progression of OA.^2^ Macroautophagy (hereafter referred to as autophagy) induction of chondrocyte has been reported to interrupt OA pathological progression.^3^, ^4^, ^5^, ^6^ Therefore, enhancement of autophagy may have chondroprotective activity in OA.^6^

Phosphoinositide‐specific phospholipases Cγ (PLCγ), including PLCγ1 and PLCγ2, could be activated by many extracellular factors to induce hydrolysis of phosphatidylinositol 4,5‐bisphosphate(PtdIns(4,5)P2) to generate two second messengers, inositol 1,4,5‐triphosphate (IP3) and diacylglycerol (DAG), triggering a series of signal pathways to regulate cellular metabolism.^7^ Other authors’ and our studies have already addressed the involvement of PLCγ1 in some chondrocyte events.^8^, ^9^, ^10^, ^11^ For example, PLCγ‐STAT1 pathway mediates apoptotic signalling by FGFR3 in the ATDC5 chondrogenic cell line.^8^ Chondrocyte migration affects tissue‐engineered cartilage integration by activating the signal transduction pathways involving Src, PLCγ1 and Erk1/2.^9^ Our previous studies showed that the disruption of PLCγ1 contributes to ECM synthesis of human OA chondrocytes.^10^ Especially, the relationship between PLCγ1 or its canonical downstream signal molecules and autophagy has been demonstrated in several studies. IP3 could activate inositol 1, 4, 5‐triphosphate receptor (IP3R) to positively or negatively regulate autophagy^12^, ^13^; DAG production is necessary for efficient autophagy of Salmonella^14^; our previous study showed that inhibition of PLCγ1 induced autophagy in colon and hepatic carcinoma cells.^15^ Hence, it is hypothesized that PLCγ1 would modulate autophagy to execute its role in OA pathological progression.

In the present study, we investigated the expression levels of ECM‐ and autophagy‐related signal molecules in rat normal or human OA chondrocytes pretreated with IL‐1β for mimicking or sustaining OA pathological condition with or without PLCγ1 inhibitor U73122 treatment. Meanwhile, the morphological changes of cartilage and related signal molecule expression levels were monitored in a rat OA model with intra‐articular injection of an adenovirus expressing ShRNA against PLCγ1(Ad‐shPLCγ1). Our findings demonstrated that PLCγ1‐driven autophagy conferred cartilage protection against OA through promoting ECM synthesis in OA chondrocytes in vitro and in vivo, involving the crosstalk between PLCγ1, AMPK, Erk and Akt.

## 2 MATERIALS AND METHODS

### 2.1 Chondrocyte isolation, culture and treatment with IL‐1β and different agents

After receiving all patient consent and in accordance with the ethical guidelines approved by the Ethics Committee of Medical School, Xiamen University, China (XMUMRE20170312), human OA chondrocytes were obtained from knee articular cartilage of 8 patients with advanced OA who were undergoing total knee replacement surgery without pretreatment of arthroscopy (Kellgren‐Lawrence (K/L) Grade III and IV, Table 1). One knee articular cartilage was from one knee in each patient. All Sprague Dawley (SD) rats were purchased from Slac Laboratory Animal Co. Ltd. (Shanghai, China). Approved by the Committee on the Ethics of Animal Experiments of Xiamen University, China (XMULAC20170354), rat chondrocytes were isolated from knee articular cartilage of 32 newborn male Sprague Dawley (SD) rats (within 0‐24 hours after birth). Joint cartilage from 1‐2 patients or 2‐3 rats was used for different types of cellular detection. As previously described,^16^ primary chondrocytes were cultured in DME/F‐12 containing 10% foetal bovine serum to 80% confluence and plated in 60‐mm cell culture dishes, followed with identification of chondrocyte property and phenotypes. Passage 1‐2 human or rat chondrocytes were pretreated by IL‐1β (20 ng/mL) for 36 hours to mimic or sustain OA pathological condition for the subsequent experiments (Figure S1).

**TABLE 1:** Information of OA patients with total knee replacement surgery for chondrocyte isolation

Age (Year)	Case	Sex		Duration of OA (year)		K/L Grade	
		M	F	≤3	>3	III	IV
55‐	2	1	1	1	1	1	1
65‐	5	1	4	2	3	1	4
75‐	1	1	0	0	1	0	1
Total	8	3	5	3	5	2	6

### 2.2 Establishing rat model of experimental OA and intra‐articular injection of an adenovirus expressing ShRNA against PLCγ1(Ad‐shPLCγ1)

A total of 110 7‐8‐week‐old male SD rats were acclimatized to the laboratory environment for one week before the experiments. Firstly, 8 of 110 rats were randomly divided into two groups, Sham (n = 4) and OA (n = 4), and the two hind knee joints of each rat in OA group underwent anterior cruciate ligament transection and medial meniscus resection (ACLT + MMx) for establishing OA model as previously described.^17^, ^18^ After four‐week post‐surgery, the 8 rats were killed, and the mixed articular cartilage of sham or OA rats (due to less articular cartilage obtained from each rat joint) was used for extracting protein and RNA of tissue, respectively. Secondly, the remaining 102 rats were randomly divided into three group, normal group (n = 8), Sham group (n = 6) and OA group (n = 88). Subsequently, the right hind knee joint of each rat in OA group (n = 88) was underwent ACLT + MMX and randomly divided into five parts with Excel‐RAND function, including OA group(n = 8), OA + 0.9% Normal Saline group(n = 8), OA + adenovirus expressing Vector (Ad‐Vector) group (n = 24), OA + adenovirus expressing ShRNA against PLCγ1‐1 (Ad‐shPLCγ1‐1) group (n = 24) and OA + Ad‐shPLCγ1‐2 group (n = 24) (Figure S2A). As described in previous studies^19^, ^20^ after the intra‐articular injection with 40 μL different agents, Ad‐shPLCγ1‐1/2, Ad‐Vector and 0.9% Normal Saline, respectively, rats were killed (Figure S2B).

### 2.3 Western blotting analysis and immunoprecipitation assay

As previously described,^15^, ^21^ human normal and OA cartilage (Tables 2 and 3), rat normal and OA model cartilage, and cultured chondrocytes were harvested, respectively, and suspended in RIPA lysis buffer, followed with centrifugation in order to obtain total protein. Protein was then subjected to SDS‐PAGE (6%‐15%) prior to transferring to a PVDF membrane (GE Healthcare, Hertfordshire, UK). The membrane was incubated with various antibodies (Table 4) as required at 4°C overnight, followed by incubation with the corresponding secondary antibodies at room temperature for 1 to 2 hours. An enhanced chemiluminescence detection kit was used to detect antibody reactivity (Pierce, Rockford, IL, USA).

**TABLE 2:** Information of OA patients with total knee replacement surgery for OA tissue protein and RNA extraction

Age (Year)	Case	Sex		Duration of OA (year)		K/L Grade	
		M	F	≤3	>3	III	IV
55‐	3	1	2	0	3	0	3
65‐	3	2	1	1	2	1	2
75‐	2	0	2	1	1	0	2
Total	8	3	5	2	6	1	7

**TABLE 3:** Information of patients for normal tissue protein and RNA extraction

Normal	Age	Diagnosis	Treatment
1	61	Fracture	THA
2	64	Fracture	THA
3	52	Trauma	Thigh amputation
4	55	Trauma	Thigh amputation

**TABLE 4:** Information of antibodies

Antibody	Manufacturer and Number	Dilution con.	Time
Collagen II	Abcam ab34712	1:100 (IHC)	4℃ over night
Collagen II	Abcam ab188570	1:1000 (WB)	4℃ over night
Aggrecan	Sigma SAB4500662‐100UG	1:100 (IHC) 1:1000 (WB)	4℃ over night
LC3B	NOVUS NB100‐2220	1:200 (IHC) 1:1000 (WB)	4℃ over night
P62 (SQSTM1)	Abcam ab56416	1:500 (IHC) 1:1000 (WB)	4℃ over night
PLCγ1	Cell Signaling technology #2822	1:100 (IHC) 1:1000 (WB)	4℃ over night
p‐PLCγ1 (Tyr783)	Cell Signaling technology #2821	1:100 (IHC) 1:1000 (WB)	4℃ over night
Erk1/2	Cell Signaling technology #4695	1:1000	4℃ over night
p‐Erk1/2 (Thr202/Tyr204)	Cell Signaling technology #9106	1:1000	4℃ over night
Akt	Cell Signaling technology #2920	1:1000	4℃ over night
p‐Akt (Ser473)	Cell Signaling technology #4051	1:1000	4℃ over night
mTOR	Cell Signaling technology #2983	1:1000	4℃ over night
p‐mTOR (Ser2448)	Cell Signaling technology #2971	1:1000	4℃ over night
ULK1	Abcam ab128859	1:1000	4℃ over night
p‐ULK1 (Ser757)	Cell Signaling technology #6888	1:1000	4℃ over night
AMPK	Cell Signaling technology #5831	1:1000	4℃ over night
p‐AMPK (Thr172)	Cell Signaling technology #2535	1:1000	4℃ over night
FAK	Cell Signaling technology #3285	1:1000	4℃ over night
p‐FAK (Tyr397)	Cell Signaling technology #3283	1:1000	4℃ over night
IP3R	Abcam ab5084	1:100 (IP) 1:1000 (WB)	4℃ over night
Bcl‐2	Cell Signaling technology #2870	1:100 (IP) 1:1000 (WB)	4℃ over night
Beclin1	Cell Signaling technology #3738	1:100 (IP) 1:1000 (WB)	4℃ over night
β‐actin	Sigma A3854	1:50 000	RT 20 min
Anti‐Mouse‐IgG (H + L) HRP	Proteintech SA00001‐1	1:25 000	RT 1 h
Anti‐Rabbit‐IgG (H + L) HRP	Proteintech SA00001‐2	1:25 000	RT 1 h
Anti‐Mouse‐IgG (H + L) Alexa Fluor 594	Thermo Fisher Scientific A21203	1:400	RT 4 h
Anti‐Rabbit‐IgG (H + L) Alexa Fluor 488	Thermo Fisher Scientific A21206	1:400	RT 4 h

Protein was mixed with Protein A&G Sepharose and corresponding antibody (anti‐IP3R, anti‐Beclin1 or anti‐Bcl‐2 antibodies) or IgG control at 4°C overnight, respectively (Table 4). Immunoprecipitation immunoblotting of the sample was then performed using anti‐IP3R, anti‐Beclin1 and anti‐Bcl‐2 antibodies as described previously.^14^, ^22^

### 2.4 Real‐time quantitative Polymerase chain reaction (qPCR)

Human normal cartilage including articular cartilage obtained from 2 patients with femoral neck fracture to undergo total hip arthroplasty (THA) and knee articular cartilage obtained from 2 patients with Trauma to undergo Thigh amputation (Table 3), human OA cartilage obtained from 8 patients with OA to undergo total knee replacement surgery (K/L Grade III and IV, Table 2), rat normal and OA model cartilage and cultured chondrocytes were harvested, respectively. The size of each human articular is 0.4‐0.6 cm × 0.4‐0.5 cm (weight 2 g), and the size of each rat articular cartilage is 0.1‐0.2 cm × 0.1‐0.2 cm (weight 0.5‐1 g).Total RNA of cartilage was extracted as described in the manufacturer's instructions (Magen, Guangzhou, China) and previous study,^23^ whereas total RNA of chondrocytes was extracted as described in the manufacturer's instructions (Bioer, Hangzhou ,China). cDNA was then synthesized with 1 μg of total RNA at 37°C for 15 minutes using a Primescript RT Master Mix Kit (Takara, Dalian, China). qPCR was then performed using a Roche LightCycler 96 (Roche, Switzerland) with a SYBR Premix Ex Taq II Kit (Takara, Dalian, China). The results were normalized to GAPDH and analysed using SDS software v2.1. The primers used for qPCR were listed in Table 5.

**TABLE 5:** Primers in qPCR

Gene name	Primer sequence (5′‐3′)
Col2a1(H) Gene ID: 1280/NM_033150.3	Forward primer: AGCAGGCGTAGGAAGGTCAT Reverse primer: AGAACTAATGGAGCAGCAAGA
Col2a1(R) Gene ID: 25412/NM_012929.1	Forward primer: TCCTAAGGGTGCCAATGGTGA Reverse primer: GGACCAACTTTGCCTTGAGGAC
ACAN(H) Gene ID: 176/XM_011521314.1	Forward primer: ACTCTGGGTTTTCGTGACTCT Reverse primer: ACACTCAGCGAGTTGTCATGG
ACAN(R) Gene ID: 58968/ J03485.1	Forward primer: TCCGCTGGTCTGATGGACAC Reverse primer: CCAGATCATCACTACGCAGTCCTC
GAPDH(H) Gene ID: 2597/NM_001289745.3	Forward primer: ATGGGGAAGGTGAAGGTCG Reverse primer: TAAAAGCAGCCCTGGTGACC
GAPDH(R) Gene ID: 24383/XM_017592435.1	Forward primer: CAAGTTCAACGGCACAGTCAAG Reverse primer: ACATACTCAGCACCAGCATCAC

### 2.5 ELISA and Dimethylmethylene blue assay

According to the manufacturer's instructions of ELISA kit from LifeSpan BioSciences (Seattle, WA, USA) and previous studies,^24^ cell culture medium was carefully collected and concentrated, followed with the detection of Collagen II level using ELISA kit. Similarly, collected culture medium was concentrated for measuring Glycosaminoglycans (GAG) level using a dimethylmethylene blue assay (DMMB) colorimetry kit from GenMed Scientifics (Wilmington, DE, USA), whereas cells were harvested, followed with extracting DNA as an inner reference of GAG, as described in the manufacturer's instructions of DMMB colorimetry and previous study.^25^

### 2.6 Immunofluorescence assay

Cells cultured on a cover glass were fixed in 4% paraformaldehyde prior to incubation with anti‐LC3B and anti‐P62 antibodies overnight.^14^, ^26^ Then, cells were incubated with anti‐Mouse‐IgG (H + L) Alexa Fluor 488 (LC3B) and anti‐Mouse‐IgG (H + L) Alexa Fluor 594 (P62) antibodies for 4 hours in the dark, respectively. Subsequently, nuclei were counterstained with 4′, 6‐diamidino‐2‐phenylindole. The stained cells were finally visualized under a laser‐scanning confocal microscope (FV1000, Olympus, Tokyo, Japan).

### 2.7 Transmission electron microscopy (TEM)

As previous described,^27^ cells were fixed in a fixative solution (2.5% glutaraldehyde, 3% paraformaldehyde and 5% sucrose in 0.1 mol/L sodium cacodylate buffer (pH 7.4)). Pelleted cells were treated with 1% OsO4‐veronal acetate prior to dehydration and embedment in Embed‐812 resin. Autophagic vacuoles were observed under a transmission electron microscope (Tecnai G2 Spirit BioTWIN, FEI Company, Hillsboro, Oregon, USA) and counted with Adobe Photoshop CS6 (San Jose, CA, USA).

### 2.8 Histopathological assay

Tissue samples were fixed in 4% paraformaldehyde, respectively, followed with dehydration and paraffin embedment. Samples were then deparaffnized in xylene and rehydrated in graded alcohols and distilled water prior to HE or Safranin O/Fast green staining. Images were scanned by Motic VM1 panoramic scanning microscope system (Motic, Hong Kong, China). After disrupted grouping, the severity of cartilage damage was semi‐quantified by two different pathologists using the Osteoarthritis Research Society International (OARSI) score system. OARSI is an OA assessment system of cartilage pathology based on six grades, which reflect depth of the lesion and four stages reflecting extent of OA over the joint surface was developed.^28^ Score 0 represents normal articular cartilage, and increasing score indicates a more biologically cartilage degeneration (a maximum possible score of 24).

### 2.9 Immunohistochemistry assay

Tissue samples were fixed in 4% paraformaldehyde, respectively, followed with dehydration and paraffin embedment. After they were deparaffnized in xylene and rehydrated in graded alcohols and distilled water, samples were incubated overnight at 4°C with anti‐Collagen II, anti‐Aggrecan, anti‐LC3B and anti‐P62 antibodies, respectively, followed by incubation with corresponding secondary antibodies (Table 4), according to the manufacturer's instructions (MAIXIN.BIO, Fuzhou, China). Diaminobenzidine was then used to visualize the immunohistochemical reaction followed by being counterstained with haematoxylin. Images were scanned with Motic VM1 panoramic scanning microscope system. As previous described,^16^ dark brown areas and cells were measured using Image J and Image‐Pro Plus 6.0 Softwares, respectively, followed with analysis using GraphPad Prism version 5 (GraphPad Software, Inc, San Diego, CA. USA).

### 2.10 Statistical analysis

Data were presented as means ± SEM of at least three independent experiments in each cell experiment and 6‐8 independent samples in each group of animal experiments. The groups were analysed for statistical significance using t test and one‐way analysis of variance (ANOVA) following Dunnett's or Tukey's post hoc test with GraphPad Prism version 6, respectively. OARSI Scoring was analysed using Kruskal‐Wallis H‐test with SPSS 19.0 software (SPSS, Chicago, IL). A value of *P* < 0.05 was regarded as statistically significant.

## 3 RESULTS

### 3.1 Suppressing PLCγ1 by its inhibitor U73122 led to the increase of Collagen II, Aggrecan and GAG levels in IL‐1β‐treated human OA and rat chondrocytes

**FIGURE 1:**
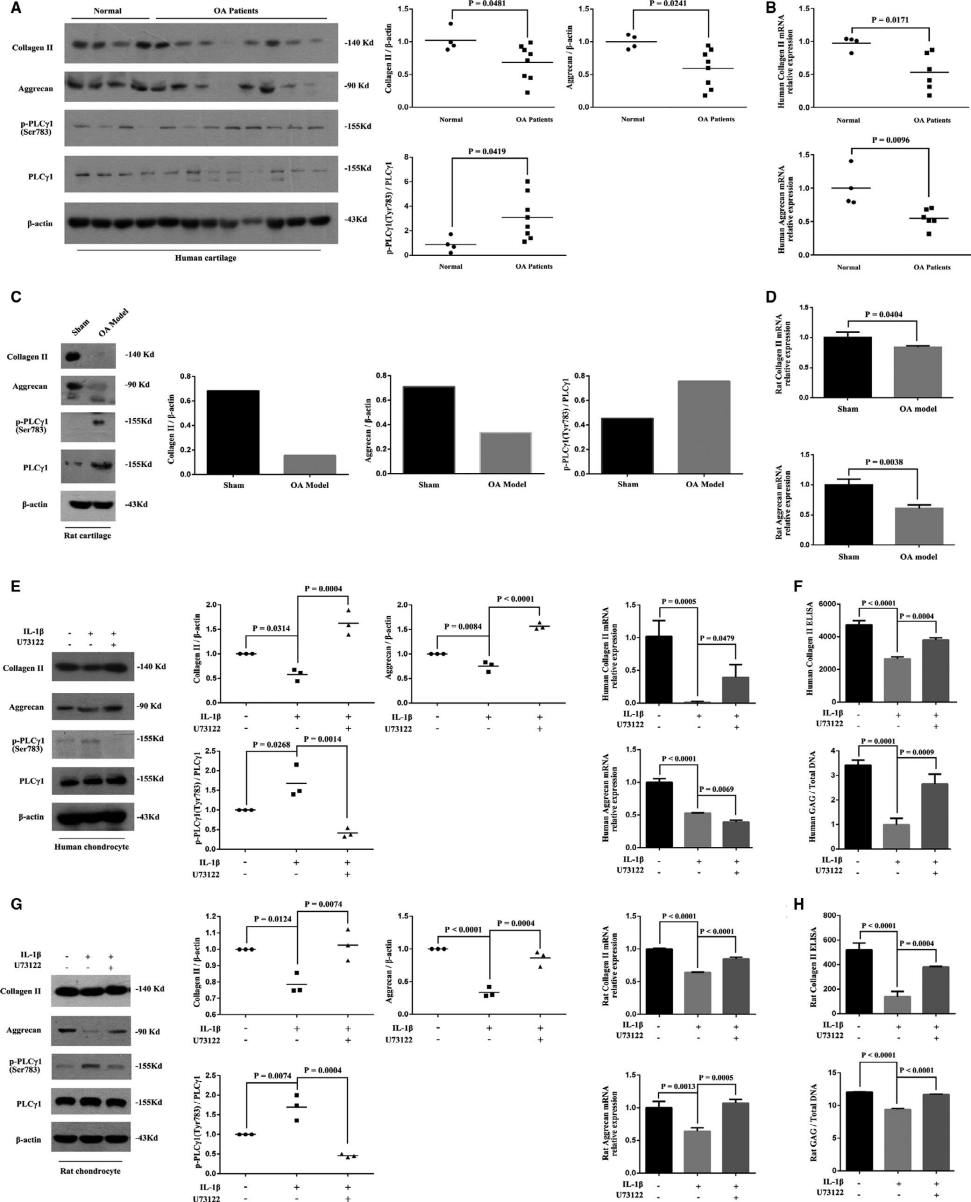
Suppressing PLCγ1 led to the increase of Collagen II, Aggrecan and GAG levels in IL‐1β‐treated human OA and rat chondrocytes. A&B, Protein of human normal and OA cartilage was extracted using RIPA buffer. The Collagen II, Aggrecan, PLCγ1, p‐PLCγ1 and β‐actin protein levels were then detected via Western blotting (A) and the Collagen II and Aggrecan mRNA levels were measured via qPCR(B). C&D, Protein of rat normal and mimicking OA cartilage (ACLT + MMx model) was extracted using RIPA buffer. The Collagen II, Aggrecan, PLCγ1, p‐PLCγ1 and β‐actin protein levels were then detected via Western blotting (C), and the Collagen II and Aggrecan mRNA levels were measured via qPCR(D). E&G, Human OA or rat chondrocytes were treated with IL‐1β (20 ng/mL) and U73122 (2 μmol/L) as described in Figure S1. The Collagen II, Aggrecan, PLCγ1, p‐PLCγ1 and β‐actin protein levels were then detected via Western blotting (left panel), and the Collagen II and Aggrecan mRNA levels were measured via qPCR (right panel). F&H, Human OA or rat normal chondrocytes were treated with IL‐1β(20 ng/mL) and U73122(2 μmol/L) as described in Figure S1. The Collagen and GAG levels in culture medium were measured with ELISA (upper panel) and Dimethylmethylene blue assay (lower panel), respectively. The values represent the means ± SEM of three independent experiments

The results of Western blotting showed that the ratio of phosphorylated PLCγ1 at Ser783 site (p‐PLCγ1)/PLCγ1, partially representing PLCγ1 activation, in human OA cartilage was higher than that in human normal cartilage, accompanied with decrease of Collagen II and Aggrecan at protein expression levels (Figure 1A, Figure S3A). Meanwhile, Collagen II and Aggrecan at mRNA levels decreased in human OA cartilage (Figure 1B, Figure S3B, vs Normal). Similar results were observed in cartilage of rat OA model (Figure 1C,D, vs Sham). Subsequently, rat normal or human OA chondrocytes were pretreated with IL‐1β for mimicking or sustaining OA pathological condition, followed with PLCγ1 inhibitor U73122 treatment, respectively. As shown in Figure 1E, IL‐1β led to a significant increase in p‐PLCγ1/PLCγ1 ratio with a significant decrease in Collagen II and Aggrecan mRNA and protein levels in human OA chondrocytes (*vs* untreated group). U73122 elevated Collagen II at protein and mRNA levels in IL‐1β‐treated human OA chondrocytes, but Aggrecan only at protein level was elevated by U73122, not at mRNA level (Figure 1E, vs IL‐1β‐treated group). Figure 1G showed that IL‐1β led to a significant increase in p‐PLCγ1/PLCγ1 ratio with a significant decrease in Collagen II and Aggrecan mRNA and protein levels (vs untreated group) and that U73122 elevated Collagen II and Aggrecan at protein and mRNA levels in IL‐1β‐treated rat chondrocytes (vs IL‐1β‐treated group). Figure 1F,H showed that U73122 also elevated the levels of Collagen II and GAG in culture medium of these chondrocytes (*vs* IL‐1β‐treated group), whereas IL‐1β led to a significant decrease in Collagen II and GAG levels (vs untreated group). Therefore, the data displayed that PLCγ1 activation linked to ECM synthesis and suppressing PLCγ1 led to the increase of Collagen II, Aggrecan and GAG levels in IL‐1β‐treated human OA and rat chondrocytes.

### 3.2 Suppressing PLCγ1 enhanced LC3B‐II/I ratio in combination with a decrease in P62 level to promote Collagen II and Aggrecan in IL‐1β‐treated human OA and rat chondrocytes, associated with the Beclin1‐IP3R‐Bcl‐2 complex and mTOR/ULK1 axis

**FIGURE 2:**
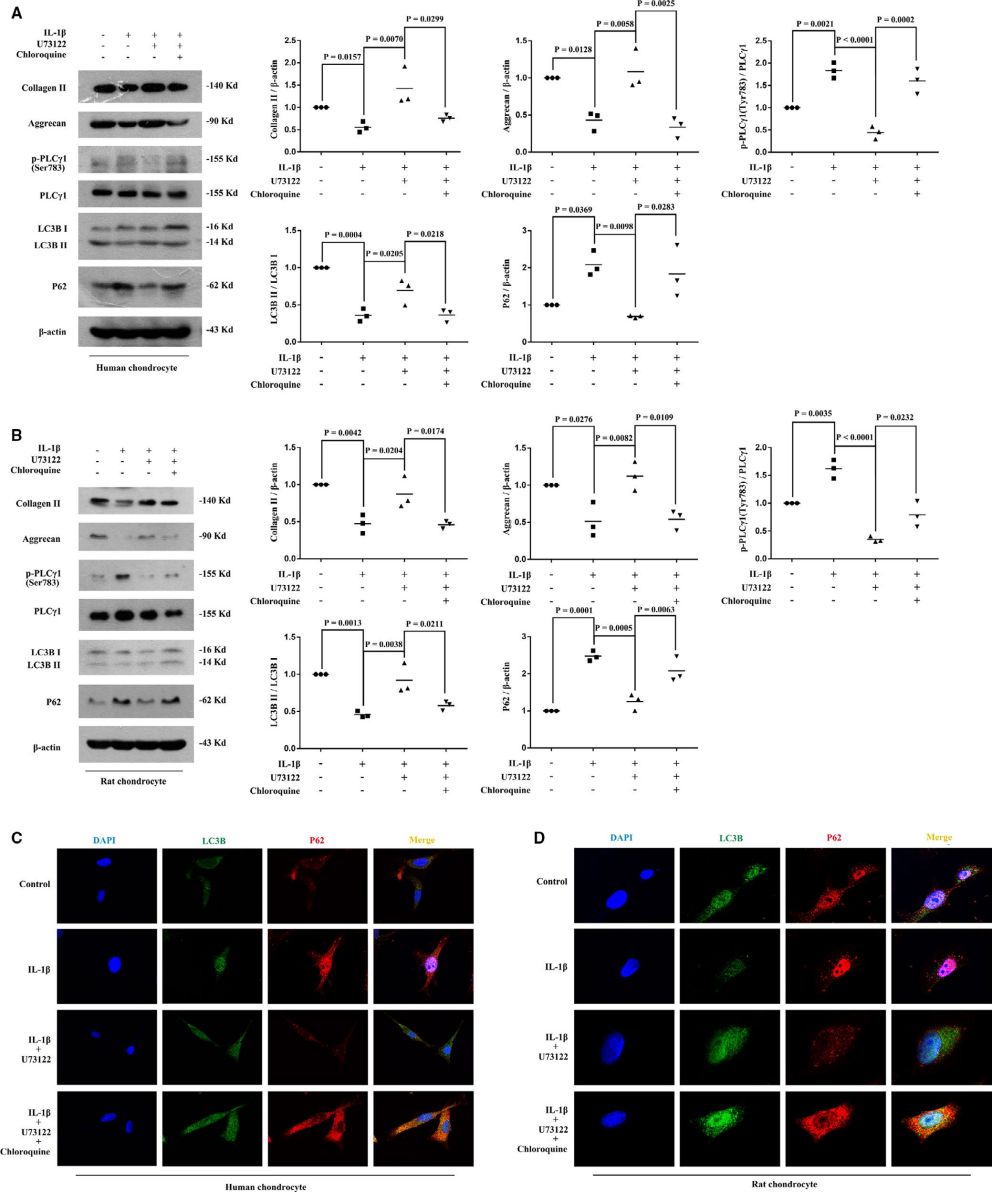
Suppressing PLCγ1 by U73122 promoted ECM synthesis through autophagy enhancement in IL‐1β‐treated human OA and rat chondrocytes. Cells were treated with IL‐1β (20 ng/mL), U73122 (2 μmol/L) and Chloroquine (50 μmol/L) as described in Figure S1. A&B, The Collagen II, Aggrecan, PLCγ1, p‐PLCγ1, LC3B, P62 and β‐actin protein levels were then detected via Western blotting. C&D, After the immunofluorescence staining was performed, the green immunofluorescence pattern of LC3B and red immunofluorescence pattern of P62 were observed under a laser‐scanning confocal microscope (original magnification × 60). The values represent the means ± SEM of three independent experiments

Compared with IL‐1β‐treated alone group, U73122 increased LC3B‐II/I ratio and reduced p62 accumulation, accompanied with increased protein expression levels of Collagen II and Aggrecan in IL‐1β‐treated human OA and rat chondrocytes, whereas autophagy inhibitor Chloroquine partially diminished its effect (Figure 2A,B, vs IL‐1β + U73122‐treated group). The observation of LC3B and P62 under a laser‐scanning confocal microscope also corroborated the occurrence of autophagy induced by U73122 in IL‐1β‐treated human OA and rat chondrocytes. Figure 2C,D showed that U73122 increased LC3B puncta (dyed green) and reduced P62 accumulation (dyed red) in cytoplasm compared with IL‐1β‐treated group, whereas Chloroquine increased the accumulation of LC3B puncta and P62, compared with IL‐1β + U73122‐treated group. Under a transmission electron microscope, it was observed that the numbers of autophagic vacuoles in IL‐1β‐treated rat chondrocytes followed with U73122 treatment were more than that in IL‐1β‐treated group, whereas IL‐1β reduced the number of autophagic vacuoles compared with untreated group (Figure S4). Similar results were observed in IL‐1β‐treated chondrocytes exposed to another autophagy inhibitor 3‐Methyladenine (Figure S5). Taken together, suppressing PLCγ1 eventually enhanced LC3B‐II/I ratio in combination with a decrease in P62 level to promote Collagen II and Aggrecan in IL‐1β‐treated human OA and rat chondrocytes.

**FIGURE 3:**
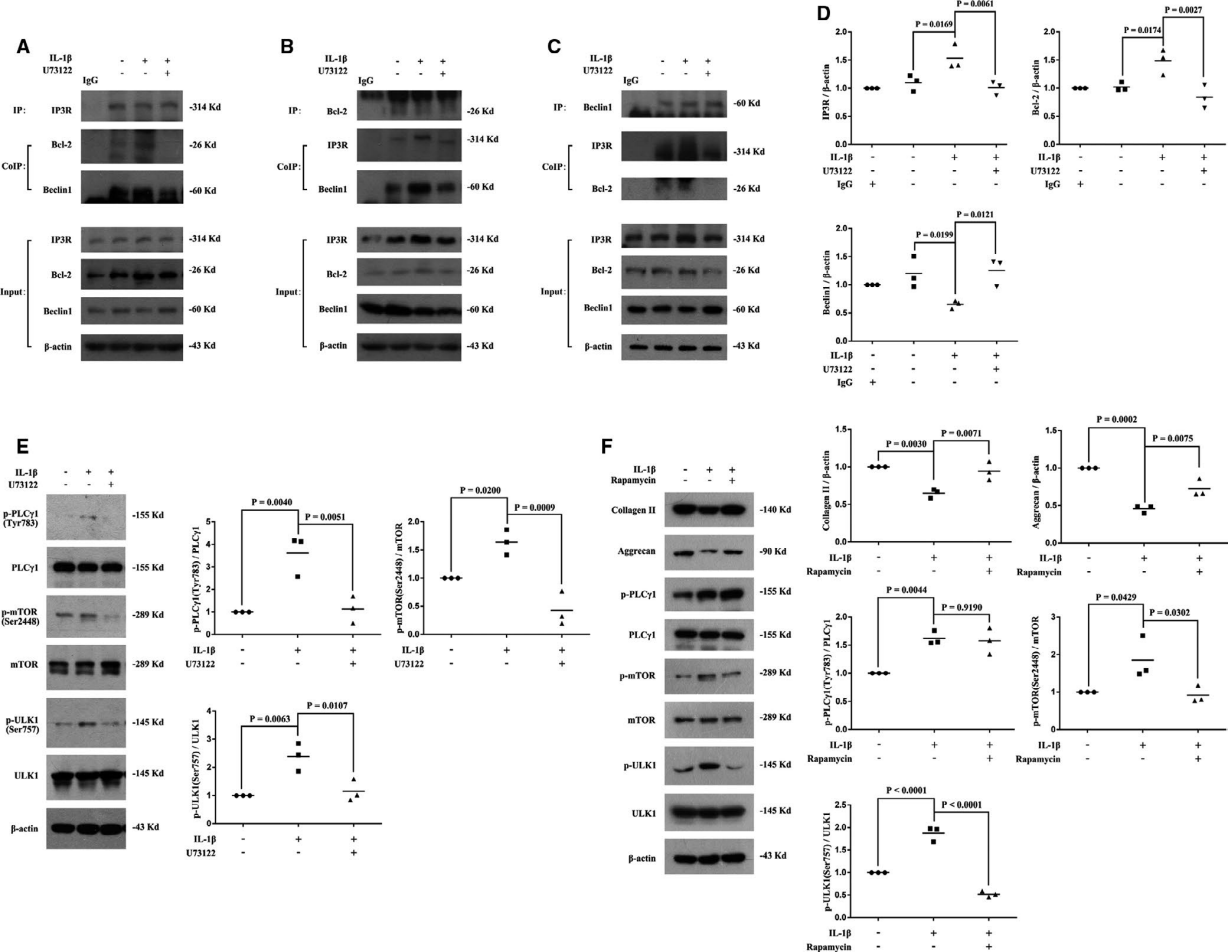
The Beclin1‐IP3R‐Bcl‐2 complex and mTOR/ULK1 axis were involved in the process of autophagy enhancement induced by PLCγ1 inhibition in IL‐1β‐treated rat chondrocyte. A‐E, Cells were treated with IL‐1β (20 ng/mL) and U73122 (2 μmol/L) as described in Figure S1. Protein extracts were subjected to immunoprecipitation with anti‐IP3R(A), Bcl‐2(B), and Beclin1(C) antibody, respectively. The immunoprecipitates were immunoblotted with IP3R, Bcl‐2 and Beclin1 antibodies. Graphs show the protein levels of IP3R, Bcl‐2 and Beclin 1 in Input (D). The PLCγ1, p‐PLCγ1, mTOR, p‐mTOR, ULK1, p‐ULK1 and β‐actin protein levels were then detected via western blotting(E). F, Cells were treated with IL‐1β (20 ng/mL) and Rapamycin (25 nmol/L) as described in Figure S1. The PLCγ1, p‐PLCγ1, mTOR, p‐mTOR, ULK1, p‐ULK1, Collagen II, Aggrecan and β‐actin protein levels were then detected via Western blotting. The values represent the means ± SEM of three independent experiments

Considering the difficulty in obtaining enough human OA chondrocytes, IL‐1β‐treated rat chondrocytes (mimicking OA chondrocytes) were used for the subsequent experiments. The results in Figure 3A,B,C (lower panels) showed that U73122 reduced IP3R and Bcl‐2 levels with increased Beclin1 level (Figure 3D, vs IL‐1β‐treated group). The results of immunoprecipitation in Figure 3A,B,C (upper panels) showed that U73122 reduced the binding of IP3R/Bcl‐2, Beclin1/Bcl‐2 and IP3R/Beclin1, compared with IL‐1β‐treated group. Additionally, compared with IL‐1β‐treated group, U73122 reduced p‐mTOR and p‐ULK1 (Ser757) levels (Figure 3E, vs IL‐1β‐treated group). Addition of mTOR inhibitor Rapamycin elevated Collagen II and Aggrecan levels without the alteration of p‐PLCγ1 (Figure 3F, vs IL‐1β‐treated group), whereas IL‐1β increased p‐PLCγ1, p‐mTOR and p‐ULK1 (Ser757) levels (Figure 3F, vs untreated group). Therefore, suppressing PLCγ1 reduced the binding of Beclin1‐IP3R‐Bcl‐2 complex and blocked the mTOR/ULK1 axis in IL‐1β‐treated rat chondrocytes.

### 3.3 Involvement of AMPK, Erk and Akt in enhanced LC3B‐II/I ratio in combination with a decrease in P62 level induced by PLCγ1 inhibitor in IL‐1β‐treated chondrocyte

**FIGURE 4:**
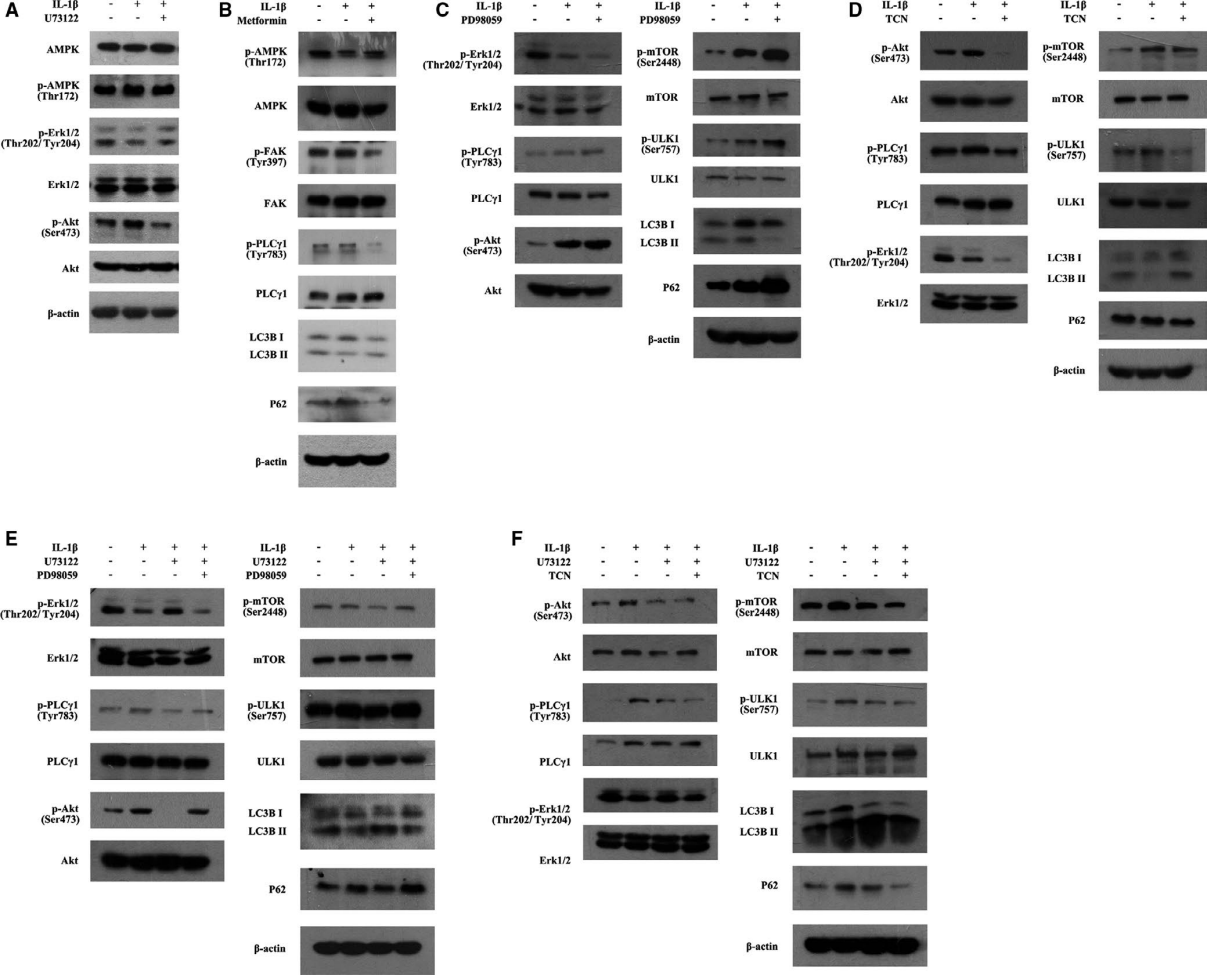
The crosstalk between PLCγ1, AMPK, Erk and Akt was involved in the process of autophagy enhancement induced by PLCγ1 inhibitor in IL‐1β‐treated chondrocytes. A, Cells were treated with IL‐1β (20 ng/mL) and U73122 (2 μmol/L) as described in Figure S1. The AMPK, p‐AMPK, Erk, p‐Erk, Akt, p‐Akt and β‐actin protein levels were then detected via western blotting. B, Cells were treated with IL‐1β (20 ng/mL) and Metformin (5 μmol/L) as described in Figure S1. The AMPK, p‐AMPK, FAK, p‐FAK, PLCγ1, p‐PLCγ1, LC3B, P62 and β‐actin protein levels were then detected via Western blotting. C, Cells were treated with IL‐1β (20 ng/mL) and PD98059 (20 μmol/L) as described in Figure S1. The Erk, p‐Erk, PLCγ1, p‐PLCγ1, Akt, p‐Akt, mTOR, p‐mTOR, ULK1, p‐ULK1, LC3B, P62 and β‐actin protein levels were then detected via Western blotting. D, Cells were treated with IL‐1β (20 ng/mL) and TCN (10 μmol/L) as described in Figure S1. The Akt, p‐Akt, Erk, p‐Erk, PLCγ1, p‐PLCγ1, mTOR, p‐mTOR, ULK1, p‐ULK1, LC3B, P62 and β‐actin protein levels were then detected via Western blotting. E, Cells were treated with IL‐1β (20 ng/mL), U73122 (2 μmol/L) and PD98059 (20 μmol/L) as described in Figure S1. The Erk, p‐Erk, PLCγ1, p‐PLCγ1, Akt, p‐Akt, mTOR, p‐mTOR, ULK1, p‐ULK1, LC3B, P62 and β‐actin protein levels were then detected via Western blotting. F, Cells were treated with IL‐1β (20 ng/mL), U73122 (2 μmol/L) and TCN (10 μmol/L) as described in Figure S1. The Akt, p‐Akt, Erk, p‐Erk, PLCγ1, p‐PLCγ1, mTOR, p‐mTOR, ULK1, p‐ULK1, LC3B, P62 and β‐actin protein levels were then detected via Western blotting. The data are representative of three independent experiments

Compared with IL‐1β‐treated group, U73122 elevated p‐AMPK level (that stimulates autophagy as cell fuel sensor) in IL‐1β‐treated rat chondrocytes (Figure 4A). Treatment of AMPK activator metformin reduced p‐FAK and p‐PLCγ1 levels, accompanied with increased LC3B‐II/I ratio in combination with a decrease in P62 level (Figure 4B, vs IL‐1β‐treated group). The results of Figure 4A also displayed the effect of U73122 on the phosphorylation of Erk(p‐Erk) and Akt (p‐Akt) in IL‐1β‐treated rat chondrocytes, expressing increased p‐Erk and decreased p‐Akt levels (vs IL‐1β‐treated group). Furthermore, MEK inhibitor PD98059 treatment elevated p‐Akt, p‐PLCγ1, p‐mTOR, and p‐ULK1 levels, accompanied with decreased LC3B‐II/I ratio in combination with an increase in P62 level (Figure 4C, vs IL‐1β‐treated group). Meanwhile, treatment of Akt inhibitor Triciribine (TCN) reduced p‐Erk, p‐PLCγ1, p‐mTOR, and p‐ULK1 levels, accompanied with increased LC3B‐II/I ratio in combination with a decrease in P62 level (Figure 4D, vs IL‐1β‐treated group). Additionally, p‐Erk and p‐Akt levels were then detected in IL‐1β/U73122‐treated chondrocytes exposed to PD98059 or TCN, respectively. As shown in Figure 4E, co‐treatment with PD98059 and U73122 attenuated the effect of U73122 on PLCγ1, mTOR and ULK1 phosphorylation, accompanied with decreased LC3B‐II/I ratio in combination with an increase in P62 level (*vs* IL‐1β + U73122‐treated group). In contrast, co‐treatment with TCN and U73122 enhanced the effect of U73122 on PLCγ1, mTOR and ULK1 phosphorylation, accompanied with increased LC3B‐II/I ratio in combination with a decrease in P62 level (Figure 4F, vs IL‐1β + U73122‐treated group). Intriguingly, co‐treatment with PD98059 and U73122 elevated p‐Akt level, but co‐treatment with TCN and U73122 reduced p‐Erk level (Figure 4E,F vs IL‐1β + U73122‐treated group). Consequently, the data indicated that the crosstalk between PLCγ1, Erk and Akt was involved in enhanced LC3B‐II/I ratio in combination with a decrease in P62 level induced by PLCγ1 inhibitor in IL‐1β‐treated chondrocyte.

### 3.4 Intra‐articular injection of Ad‐shPLCγ1 ameliorated cartilage damage in a rat OA model

**FIGURE 5:**
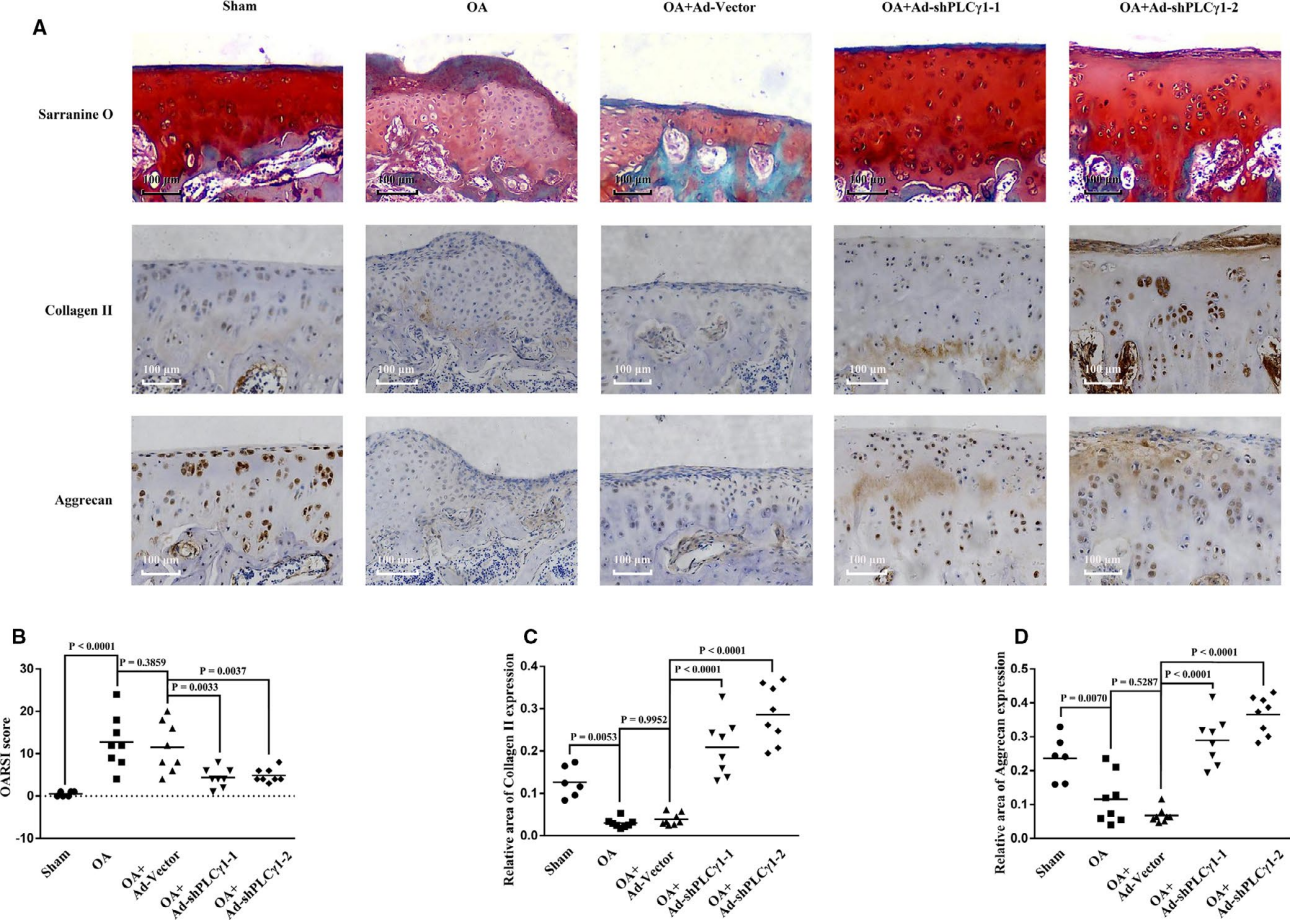
Intra‐articular injection of Ad‐shPLCγ1 ameliorated cartilage damage in a rat OA model. A, Representative images of Safranin O/Fast green staining and immunohistochemistry assay for Collagen (1:100) and Aggrecan (1:1000) (original magnification × 10), respectively. B, Graph shows the OARSI score in different injected groups. C, Graph shows the relative area expressing Collagen II in different injected groups. D, Graph shows the relative area expressing Aggrecan in different injected groups. The values represent the means ± SEM

To better mimic environment of knee joint, a rat OA model was induced by ACLT + MMx and intra‐articularly injected of Ad‐shPLCγ1. Based on the results of Safranin O/Fast green staining (upper panel in Figure 5A), the severity of cartilage damage was then scaled using OARSI grade score system.^28^ The OARSI Scores in OA + Ad‐shPLCγ1‐1 and OA + Ad‐shPLCγ1‐2 groups were lower than that in OA + Ad‐Vector group, without difference between OA and OA + Ad‐Vector groups (Figure 5B). Furthermore, Figure 5C,D displayed that Collagen II and Aggrecan expression levels in OA + Ad‐shPLCγ1‐1 and OA + Ad‐shPLCγ1‐2 groups were higher than that in OA + Ad‐Vector group. Therefore, the data indicated that intra‐articular injection of Ad‐shPLCγ1ameliorated cartilage damage in rat OA model.

### 3.5 Intra‐articular injection of Ad‐shPLCγ1 enhanced LC3B level in combination with a decrease in P62 level in a rat OA model

**FIGURE 6:**
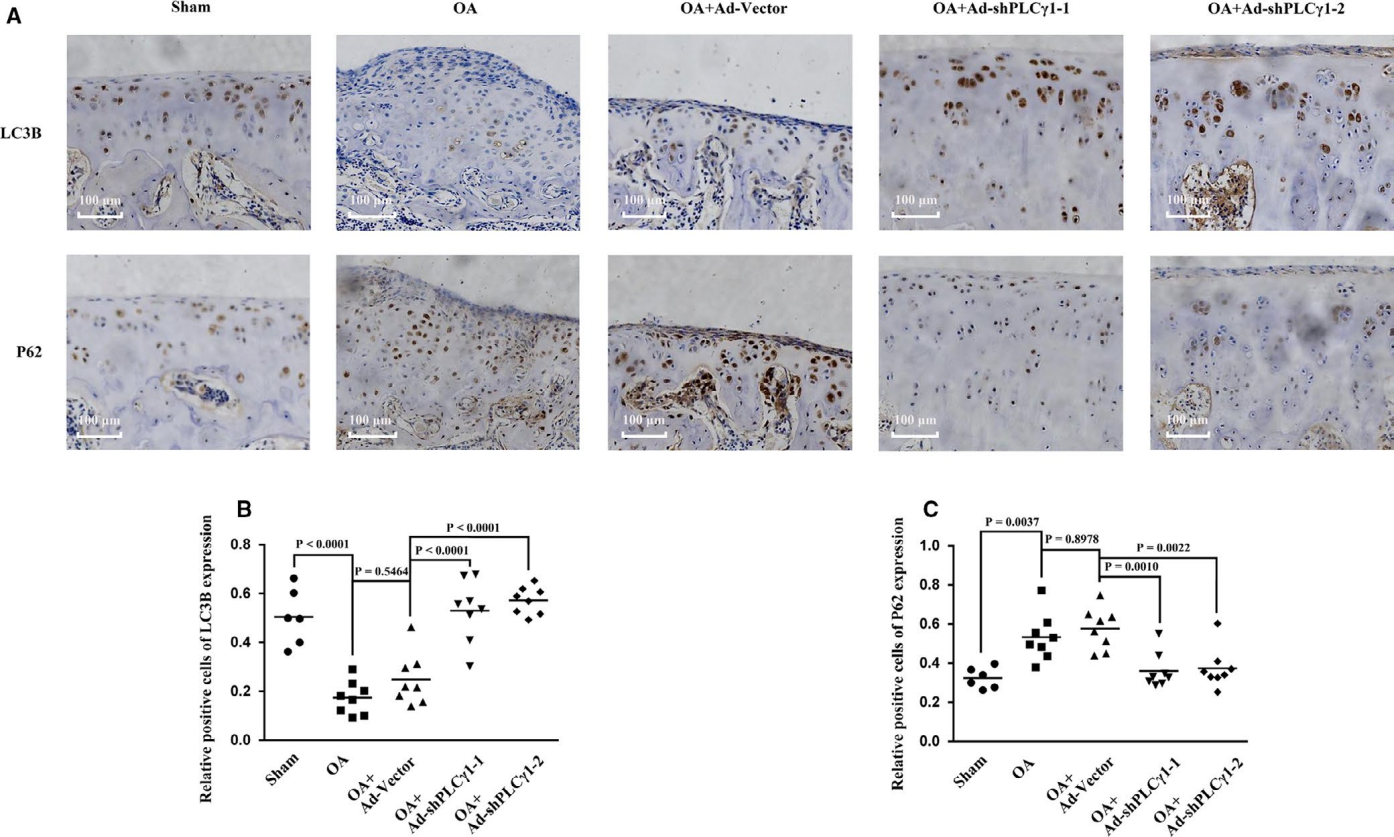
Intra‐articular injection of Ad‐shPLCγ1 enhanced LC3B level in combination with a decrease in P62 level in a rat OA model. A, Representative images of immunohistochemistry assay for LC3B (1:800) and P62 (1:100) (original magnification × 10). B, Graph shows the positive cells expressing LC3B in different injected groups. C, Graph shows the positive cells expressing P62 in different injected groups. The values represent the means ± SEM

The expression levels of LC3B and P62 were detected in a rat OA model using immunohistochemistry assay (Figure 6A). LC3B reduced in cartilage of OA group (Figure 6B, vs Sham group]. Intra‐articular injection of an adenovirus expressing shPLCγ1‐1 or shPLCγ1‐2 led to an increase of LC3B level in OA + Ad‐shPLCγ1‐1 and OA + Ad‐shPLCγ1‐2 groups (Figure 6B, vs OA + Ad‐Vector group). In contrast, P62 increased in cartilage of OA group (Figure 6C, vs Sham group). Intra‐articular injection of Ad‐shPLCγ1 decreased P62 level in OA + Ad‐shPLCγ1‐1 and OA + Ad‐shPLCγ1‐2 groups (Figure 6C, vs OA + Ad‐Vector group). Consequently, intra‐articular injection of Ad‐shPLCγ1 enhanced LC3B level in combination with a decreased in P62 level in a rat OA model.

## 4 DISCUSSION

In the present study, we demonstrated that PLCγ1 inhibition by U73122 enhanced Collagen II, Aggrecan and GAG levels through increasing of LC3B‐ II/I ratio in combination with a decrease in P62 level in IL‐1β‐treated human OA and rat chondrocytes, suggesting that suppressing PLCγ1 could enhance autophagy to promote ECM synthesis. Furthermore, both the dissociation of Beclin1 from Beclin1‐IP3R‐Bcl‐2 complex and the blockade of mTOR/ULK1 axis might contribute to enhanced autophagy induced by PLCγ1 inhibition in IL‐1β‐treated human OA and rat chondrocytes. The crosstalk between PLCγ1, AMPK, Erk and Akt signal molecules was involved in the process of autophagy enhancement induced by PLCγ1 inhibition. Additionally, the cartilage protection of intra‐articular injection of Ad‐shPLCγ1, exhibiting decreased OARSI scores and increased levels of Collagen II, Aggrecan and proteoglycans, accompanied with increased LC3B level in combination with a decrease in P62 level, was also testified in a rat OA model, suggesting that PLCγ1 depletion by Ad‐shPLCγ1 ameliorated cartilage damage, accompanied with autophagy enhancement. Therefore, suppressing PLCγ1 could execute an efficacious protection on cartilage against OA through enhancing autophagy to promote ECM synthesis in IL‐1β‐treated chondrocytes or a rat OA model.

Consistent with previous studies,^10^, ^11^, ^29^ our findings displayed that PLCγ1 was expressed at elevated level in cartilage of human OA and rat OA model, as well as both human and rat OA chondrocytes. Furthermore, the data that PLCγ1 inhibitor increased Collagen II and Aggrecan at protein and mRNA levels in IL‐1β‐treated human OA and rat chondrocytes and intra‐articular injection of Ad‐shPLCγ1 decreased OARSI scores and increased levels of Collagen II, Aggrecan and proteoglycans in rat OA model indicated the cartilage protection of PLCγ1 inhibition against OA. Based on these results, we suggest that suppressing PLCγ1 could enhance autophagy to promote ECM synthesis, gradually being a potential target for OA therapy.

Autophagy activation could be identified as a cartilage protective factor.^3^, ^4^, ^5^, ^6^ Here, we found that PLCγ1 inhibitor U73122 enhanced LC3B‐II/I ratio in combination with a decrease in P62 level to promote ECM synthesis in IL‐1β‐treated human OA and rat chondrocytes. Subsequently, autophagy inhibitors Chloroquine or 3‐Methyladenine blocked the promoting effect of U73122 on ECM synthesis. Meanwhile, intra‐articular injection of Ad‐shPLCγ1 ameliorated cartilage damage accompanied with increased LC3B level in combination with a decrease in P62 level in a rat OA model. Hence, these results support the notion that PLCγ1 inhibitor U73122 enhanced autophagy to promote ECM synthesis, consistent with our previous study in colon and hepatic carcinoma cells.^15^ Like previous studies in other cell types,^12^, ^13^, ^15^ PLCγ1 inhibition induced autophagy through accelerating the dissociation of Beclin1 from Beclin1‐IP3R‐Bcl‐2 complex in IL‐1β‐treated rat chondrocytes, because U73122 reduced IP3R and Bcl‐2 levels and elevated Beclin1 level, accompanied with decreased binding of IP3R/Bcl‐2, Beclin1/Bcl‐2 and IP3R/Beclin1. Additionally, our findings showed that PLCγ1 inhibition reduced p‐mTOR and p‐ULK1 in IL‐1β‐treated rat chondrocytes, indicating the involvement of mTOR/ULK1 axis in enhanced autophagy induced by PLCγ1 inhibition. Similar role of activated mTOR/ULK1 axis in autophagy has been impressively reported in previous studies.^15^, ^30^, ^31^, ^32^ Consequently, PLCγ1 inhibition by U73122 eventually enhanced autophagy in IL‐1β‐treated chondrocytes, promoting ECM synthesis, in which both the dissociation of Beclin1 from Beclin1‐IP3R‐Bcl‐2 complex and blockade of the mTOR/ULK1 axis were involved.

PLCγ1 has been reported to be positively modulated by activated FAK,^33^ and the latter is demonstrated to negatively regulate autophagy.^34^, ^35^ Additionally, FAK might be an AMPK substrate to regulate autophagy.^36^, ^37^ Our previous study showed that activated AMPK triggered autophagy via blockade of the FAK/PLCγ1 axis in HCT116 and HepG2 cells.^15^ Hence, it is speculated that both AMPK and FAK might be involved in enhanced autophagy induced by PLCγ1 inhibition in IL‐1β‐treated chondrocytes. Our findings that PLCγ1 inhibitor U73122 elevated p‐AMPK level and AMPK activator metformin reduced p‐FAK and p‐PLCγ1 levels, accompanied with increased LC3B‐II/I ratio in combination with a decrease in P62 level in IL‐1β‐treated chondrocytes, indicated that AMPK negatively regulated the FAK/PLCγ1 axis to enhance autophagy, suggesting that the FAK/PLCγ1 axis might be a potential downstream effect of AMPK activation‐dependent autophagy in IL‐1β‐treated chondrocytes.

Fan et al have illustrated that the up‐regulation of Erk by Progranulin elevates the levels of anabolic biomarkers in degenerative human chondrocyte.^38^ Our previous study showed that Erk activation by morroniside promoted matrix synthesis in human OA chondrocytes.^18^ These studies highlight a beneficial role of Erk in OA therapy. Our findings that U73122 promoted ECM synthesis accompanied with increasing p‐Erk level also indicated the beneficial role of Erk activation for OA therapy. In addition, Erk activation could promote autophagy in ovarian cancer cells,^39^ hepatocellular carcinoma cells^40^ and LPS‐treated rat chondrocytes.^41^ Consistent with these studies, our observation that PD98059 elevated p‐PLCγ1, p‐mTOR and p‐ULK1 levels and reduced LC3B‐II/I ratio in combination with an increase of P62 level also indicated positive regulation of Erk activation in the process of enhanced autophagy induced by PLCγ1 inhibition. Therefore, we suggest that PLCγ1 inhibition could enhance autophagy through up‐regulating p‐Erk level, resulting in increased ECM synthesis in IL‐1β‐treated rat chondrocytes.

The intracellular signalling pathway PI3K/Akt has been demonstrated to be involved in both cellular and ECM alterations of chondrocyte.^42^ However, there is not yet a consensus on the role of Akt activation in chondrocyte metabolism.^42^, ^43^ Akt is always considered to be an autophagy suppressor,^43^ although some studies support opposite view.^44^ Here, we observed that U73122 promoted ECM synthesis accompanied with reduced p‐Akt level, reflecting a negative role of Akt for OA therapy; that TCN reduced p‐PLCγ1, p‐mTOR and p‐ULK1 levels, accompanied with alterations of LC3B and P62, also reflecting a negative role of Akt in the process of enhanced autophagy induced by PLCγ1 inhibition. Consequently, PLCγ1 inhibition could enhance autophagy through decreasing p‐Akt level, resulting in increased ECM synthesis in IL‐1β‐treated rat chondrocytes.

Besides, the above‐mentioned results illustrated a reciprocal suppression between PLCγ1 and Erk in OA chondrocyte and a reciprocal promotion between PLCγ1 and Akt in OA chondrocyte, which has been demonstrated in previous studies.^9^, ^11^, ^29^ Our further data that PD98059 elevated p‐Akt level and TCN reduced p‐Erk level accompanied with alterations of LC3B and P62 indicated that Erk negatively regulated Akt activation and Akt positively regulated Erk activation in IL‐1β‐treated rat chondrocytes exposed to U73122. The opposing roles of Erk and Akt in both autophagy and ECM synthesis have been already addressed in malignant glioma cells^45^ and human OA chondrocytes.^46^ Hence, the differential regulation of Erk and Akt by PLCγ1 could be involved in the process of autophagy enhancement induced by PLCγ1 inhibition to promote ECM synthesis in IL‐1β‐treated rat chondrocytes.

A limitation to our study is the lack of evaluating the effect of intra‐articular injection of Ad‐shPLCγ1 on the other components of knee joint in animal experiment. Another limitation in this study is that it is necessary to precisely target PLCγ1 in chondrocyte to inhibit its expression, minimizing damage to other cells in articular cavity using CRISPR‐Cas9 technique.

## 5 CONCLUSION

**FIGURE 7:**
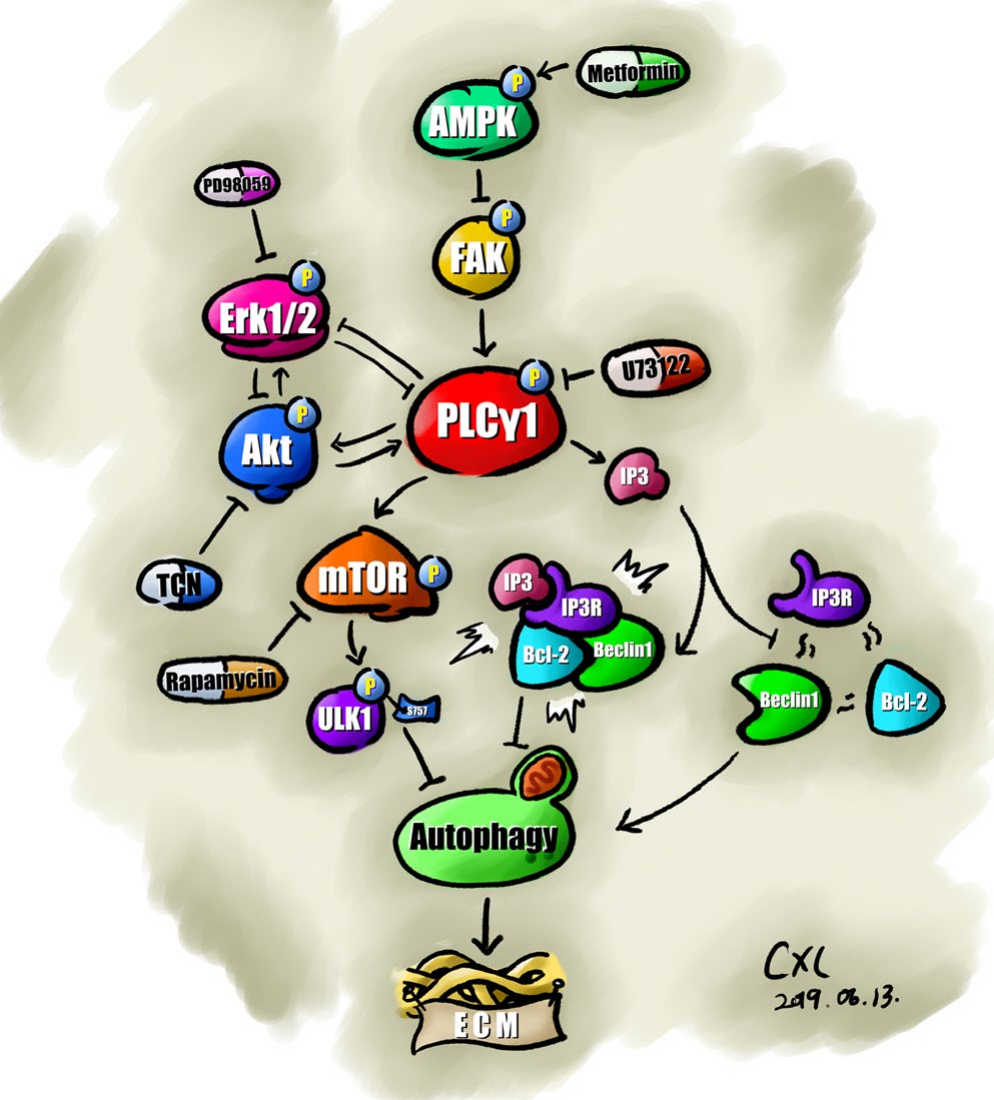
Schematic diagram illustrating the effect and regulatory mechanism of PLCγ1 inhibition on ECM synthesis through enhancement of autophagy in OA chondrocytes

Our findings demonstrated that PLCγ1 inhibition by its inhibitor U73122 promoted ECM synthesis through enhancement of autophagy in IL‐1β‐treated human OA and rat chondrocytes, in which both the dissociation of Beclin1 from Beclin1‐IP3R‐Bcl‐2 complex and the blockade of mTOR/ULK1 axis might be involved. Meanwhile, the crosstalk between PLCγ1, AMPK, Erk and Akt could modulate the process of autophagy enhancement induced by PLCγ1 inhibition. Furthermore, PLCγ1 depletion by shRNA/PLCγ1 conferred cartilage protection against OA through autophagy enhancement in a rat OA model, with an implication that targeting PLCγ1 might be a potential therapeutic approach for OA (Figure 7).

## CONFLICT OF INTEREST

The authors confirm that there are no conflicts of interest.

## AUTHOR CONTRIBUTION

**Xiaolei Chen:** Conceptualization (equal); Data curation (lead); Investigation (lead); Methodology (lead); Validation (lead); Writing‐original draft (equal). **Yue Wang:** Formal analysis (supporting); Investigation (supporting); Methodology (supporting). **Ning Qu:** Investigation (supporting); Supervision (supporting). **bing zhang:** Conceptualization (lead); Project administration (supporting); Supervision (supporting); Writing‐original draft (lead); Writing‐review & editing (lead). **Chun Xia:** Conceptualization (lead); Funding acquisition (lead); Project administration (lead); Resources (lead); Supervision (lead); Writing‐review & editing (lead).

## Supporting information

Fig S1

Fig S2

Fig S3

Fig S4

Fig S5

## Data Availability

The data support the findings of this study are available in the supplementary material of this article.
