# Supplementary figures and images for: PLCγ1 inhibition‐driven autophagy of IL‐1β‐treated chondrocyte confers cartilage protection against osteoarthritis, involving AMPK, Erk and Akt
Source: J Cell Mol Med. 2020 Dec 28;25(3):1531–45. doi: 10.1111/jcmm.16245 (PMC7875910; doi:10.1111/jcmm.16245)

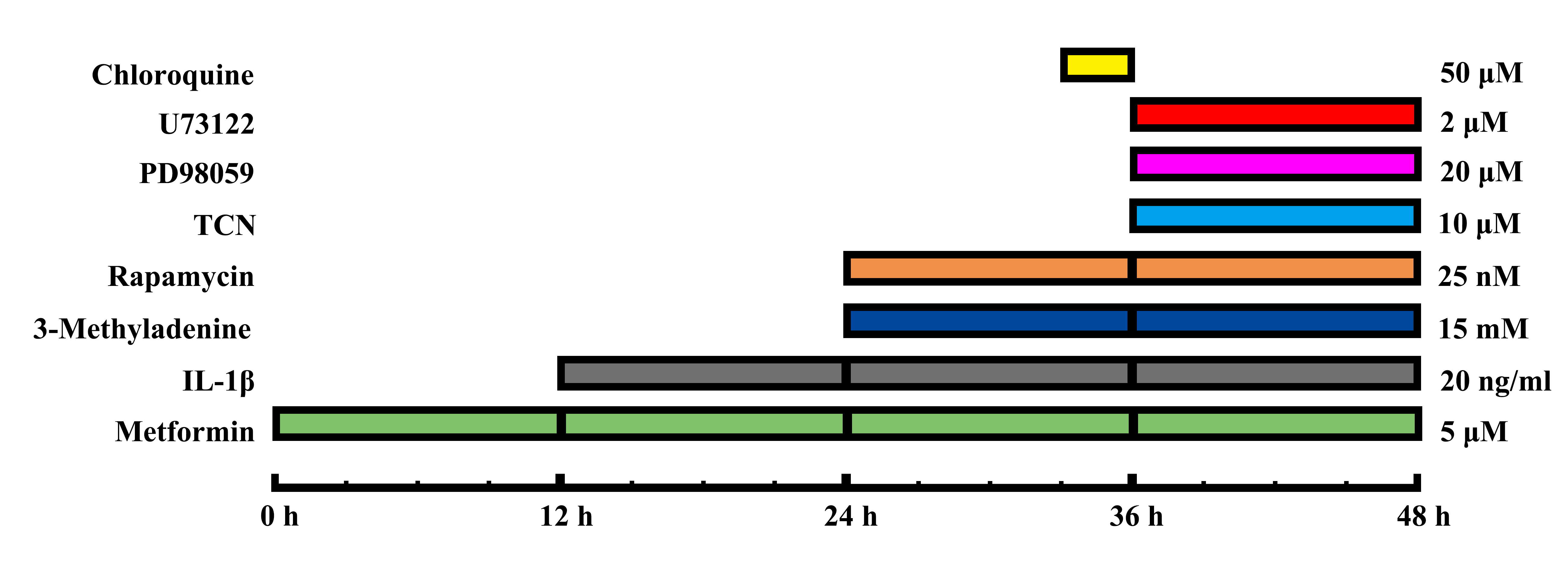

Supplement: Supplementary file 1 — Fig S1 [file JCMM-25-1531-s001.jpg]

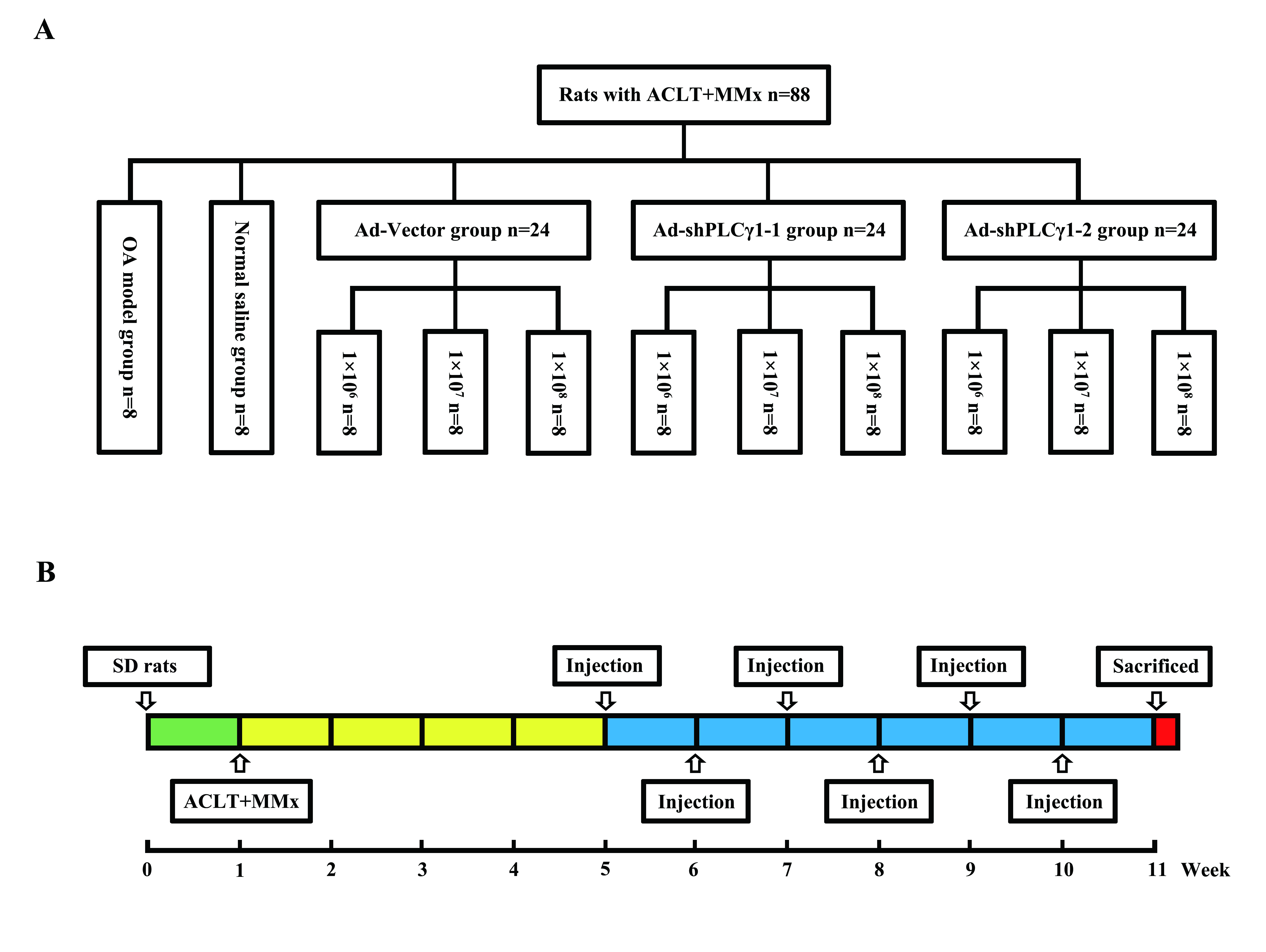

Supplement: Supplementary file 2 — Fig S2 [file JCMM-25-1531-s002.jpg]

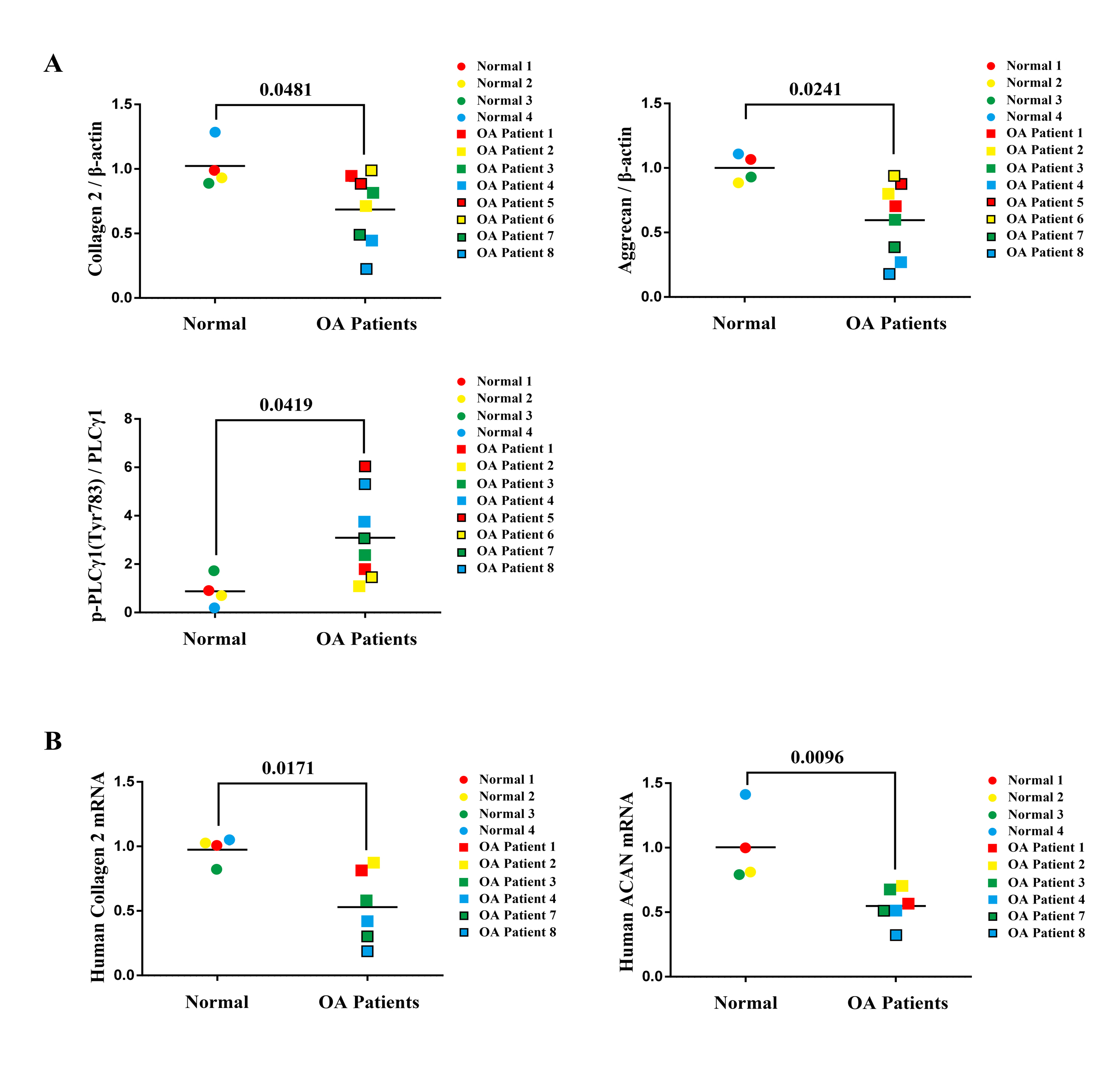

Supplement: Supplementary file 3 — Fig S3 [file JCMM-25-1531-s003.jpg]

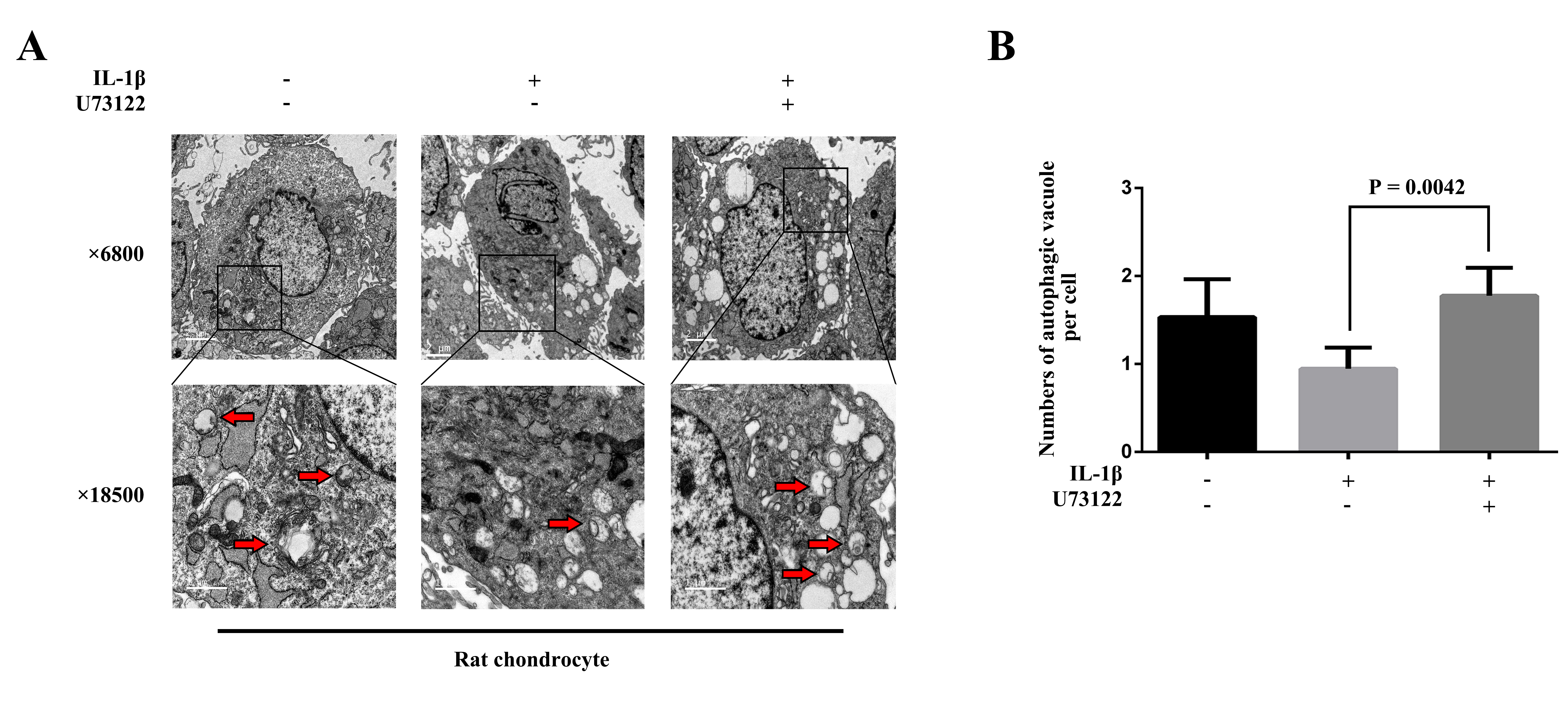

Supplement: Supplementary file 4 — Fig S4 [file JCMM-25-1531-s004.jpg]

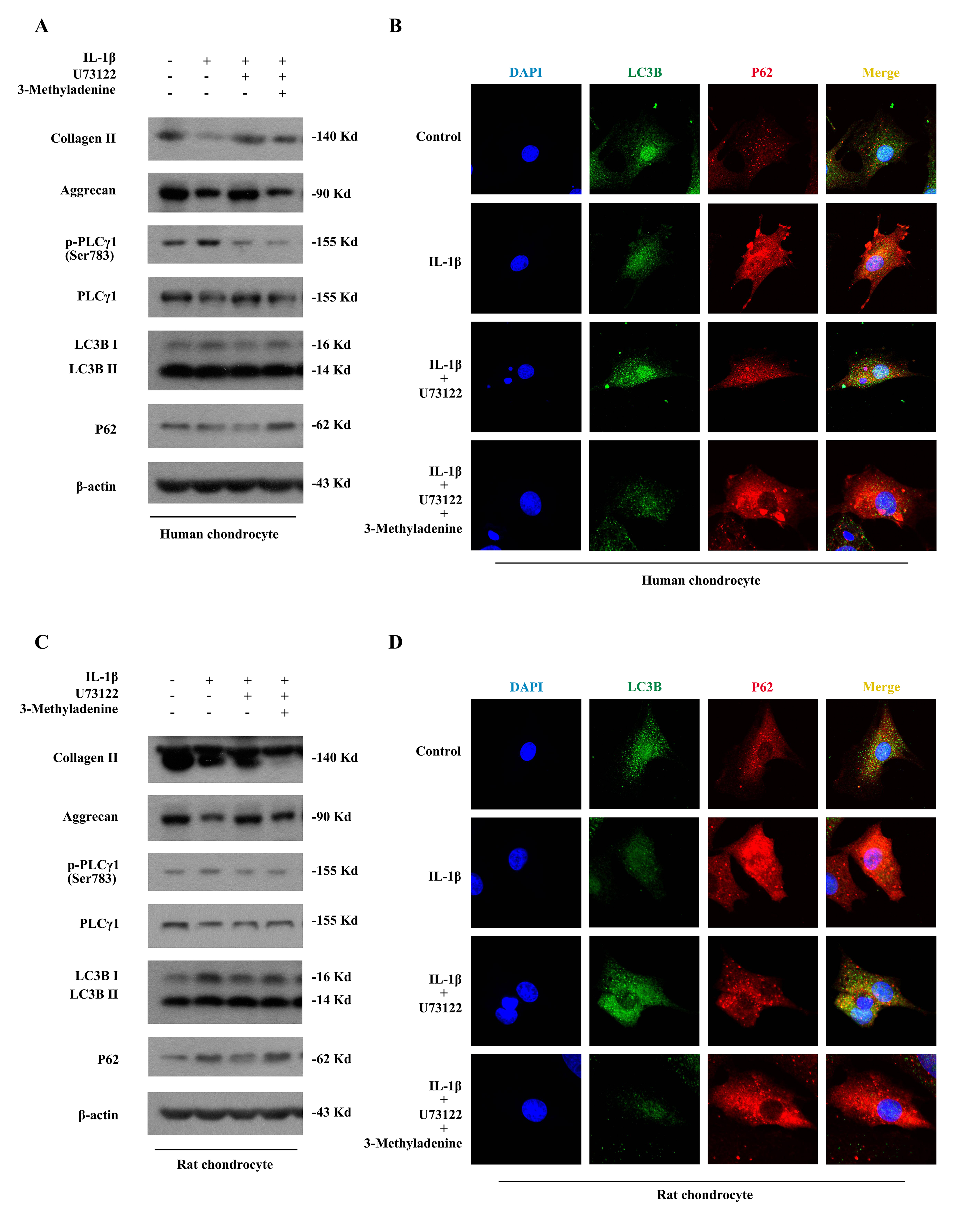

Supplement: Supplementary file 5 — Fig S5 [file JCMM-25-1531-s005.jpg]
